# A Case of Subpericranial Cephalæmatoma

**Published:** 1885-10

**Authors:** John. D. S. Davis

**Affiliations:** Birmingham, Ala.


### CASE OF SUBPERICRANIAL CEPHALÆMATOMA.

BY JOHN D. S. DAVIS, M. D., BIRMINGHAM, ALA.

On the 10th of June last, I was called upon by Wesley G. to treat his baby, a male, ten days old. On entering the room I found the baby lying on a soft pillow, breathing stertorous, with pupils contracted and an inability to nurse. There was retention of urine. My attention was called to an unusually large head, which I found to be due to a large fluctuating tumor, which covered both parietal bones and the squamous portion of the temporal bone of the right side. The mother was a primipara and was attended by a skilled midwife, who told me that there was a vertex presentation, and that labor did not last an hour. The child weighed eight pounds at birth. The mother did well, and so did the child until the sixth day, when a tumor began to make its appearance on the right side of the head. It continued to increase in size without bad effect until the tenth day, the midwife observed that there was suppression of the urine, inability to nurse, contracted pupils and slow breathing. I examined the tumor thoroughly and found it to be free over bones mentioned, soft at the base and center, with no pulsations, which was convincing to me that the extravasation was external to the bones only. Yet, when I knew of the slow breathing, contracted pupils, suppressed urine, etc., I was led to believe that one of two conditions existed: That there was an extravasation of fluid between the bone and *dura mater*, with external extravasation; or, that I had an external extravasation with an undeveloped condition of the cranial bones, allowing the pressure of fluid on the brain. The whole aspect of the little sufferer did but tract me to believe that death would soon be inevitable if something was not done for relief. And hence, I was but a few minutes in preparation for operating the tumor. I introduced an aspirating needle into the tumor over the region of the right parietal eminence and drew out two and one-half ounces of venous blood. On introducing the needle into right side of the tumor, I did not believe that the contents of both sides would be drawn off, until the tumor began to diminish in size, showing that the tumor had one large cavity and was not double, as is usual with this class of tumors, for we know that the periosteum is, as a rule, firmly adherent at the sutures, and does not usually allow fluid to pass beyond the suture of bone over which it may occur; and the fact that the fluid is not allowed to pass beyond these boundaries converts these large tumors into multiple tumors, the fluid of each being confined to the bone over which we have the rupture of vessels. This is a distinguishing feature in these cases that was wanting in this particular case, as was shown by the operation.

This operation was followed with almost immediate relief, for the bad symptoms began to disappear, and at the expiration of four hours the baby could nurse, and the breathing was free and without difficulty. Compression was applied by means of the rubber cap, and the head rested on a rubber air-pillow. The compression was retained until the following morning, when I found the child rapidly going into the condition in which I first found it. The respiration, pupils and an inability to nurse led me to believe that there was yet a hemorrhage going on, and that my compress would not allow room for it. And hence the pressure was brought upon the brain. I took off the compress, and had the pleasure of seeing my little patient relieved for the time being. The child continued better until the fourth day, when I found that the tumor had refilled, and that it was necessary to operate again. This I did, and drew off two ounces of venous blood. I found it necessary to operate three times more, getting one and one-half ounces of blood on June 17th, one and one-fourth ounces June 21st, and one ounce June 28th.

This infant weighed eight pounds, and within the first twenty-eight days of its life it lost by the operations eight and one-fourth ounces of blood.

With this case, I wish to call attention to a case of *sub-pericranial cephalœmatoma*, reported by myself in Vol. VII of *The Weekly Medical Review* of May 12th, 1883, page 335. It was a baby weighing six pounds at birth. The tumor made its appearance on the fourth day, and soon covered the right parietal bone and squamous portion of right temporal bone. I aspirated that tumor four times within the first twenty-five days of its life, and drew off in all seven and one-half ounces of blood.

Both cases resulted well, and are now living and in good health. Both of these cases were treated with the compress with no avail, but, to the contrary, with a renewal of all the bad symptoms, which I so much desired to relieve. I account for this from an imperfect development of the cranial bones, which were too soft and undeveloped to get the pressure that is necessary in treating these cases by compress without injury to the brain.

